# RICTOR Prevents Decidualization Disorder in Women With Advanced Maternal Age via Regulating FoxO1 Nuclear Export

**DOI:** 10.1111/acel.70667

**Published:** 2026-08-21

**Authors:** Yifeng Lin, Jiwei Sun, Yue Ying, Dan Li, Yuewen Ying, Xilin Shen, Xiao Sun, Dan Zhang, Yiqing Wu, Runju Zhang

**Affiliations:** ^1^ Key Laboratory of Reproductive Genetics (Ministry of Education) and Department of Reproductive Endocrinology, Women's Hospital, School of Medicine Institute of Medical Genetics and Development, Zhejiang University Zhejiang China; ^2^ Zhejiang Provincial Birth Defect Control and Prevention Research Center Zhejiang Hangzhou China; ^3^ Center for Reproductive Medicine, the First Affiliated Hospital, School of Medicine Zhejiang University Zhejiang Hangzhou China

**Keywords:** decidualization disorder, FoxO1, RICTOR, women with advanced maternal age

## Abstract

Miscarriage and abnormal embryonic development in women of advanced maternal age (AMA) are often associated with impaired decidualization. mTORC2 is an evolutionarily conserved protein kinase. As a core component of mTORC2, RICTOR has been implicated in nutrient sensing and is closely linked to implantation disorders; however, its role in regulating age‐related decidualization disorders remains unreported. In this study, we identified pronounced decidualization defects in AMA foster mice, accompanied by a significant reduction in RICTOR in the decidual tissues of AMA women. Uterine‐specific genetic deletion of *Rictor* in mice resulted in pregnancy loss and impaired decidualization. Notably, *Rictor* knockdown led to attenuated Akt3–FoxO1 signaling and impaired nuclear export of FoxO1. Molecular and histological analyses demonstrated that the specific RICTOR activator MHY1485 effectively rescued decidualization defects in vivo and in vitro. These findings indicate that RICTOR plays an essential role in the aging decidual microenvironment and may serve as a potential biomarker for uterine stromal cell decidualization. Furthermore, therapeutic activation of RICTOR represents a promising strategy to counteract senescent decidual impairment in AMA women and improve pregnancy outcomes.

## Graphical abstract

Advanced maternal age impairs uterine decidualization via downregulated RICTOR‐ATK3‐FoxO1 signaling. The RICTOR agonist MHY1485 rescues decidual defects, offering a therapy for age‐related pregnancy failure.

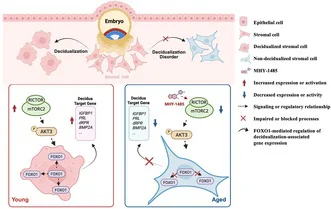

## 1 Introduction

Rising levels of educational and professional attainment have contributed to a global increase in maternal age at childbearing (Monaghan et al. 2020). Advanced maternal age (AMA), typically defined as 35 years or older at the time of delivery (de Jongh et al. 2015; Geiger et al. 2021), is consistently associated with a greater risk of reproductive challenges. These challenges include increased risks of infertility, implantation failure, miscarriage, as well as other adverse pregnancy outcomes (Zhang et al. 2022; Zhou et al. 2023).

Although diminished ovarian reserve and declining oocyte quality are well‐recognized factors in age‐related fertility decline, growing evidence underscores the critical contribution of endometrial dysfunction (Pathare et al. 2023). Clinical studies involving preimplantation genetic testing (PGT) cycles and egg donation programs indicate that even with euploid embryo transfer or oocytes from young donors, pregnancy outcomes among AMA women remain suboptimal (Harton et al. 2013; Sabbagh et al. 2025; Yeh et al. 2014). These findings underscore the importance of the endometrial environment in reproductive aging.

Embryo implantation relies on decidualization, a progesterone‐dependent process in which endometrial stromal cells differentiate into decidual cells that support early embryonic development (Cha et al. 2012). During decidualization, endometrial stromal cells undergo morphological and functional changes under the influence of progesterone, resulting in their transformation into decidual stromal cells (Cha et al. 2012). This process provides essential support and nutrition for the embryonic development following implantation. Impaired decidualization is associated with implantation failure and early pregnancy loss (Cha et al. 2012). Recent studies have identified that senescence of endometrial cells contributes to impaired endometrial decidualization and disrupts interactions with trophoblast cells. Decidualization defects observed in aged mice suggest a conserved mechanism that may also be relevant to humans. Specifically, the senescence of endometrial stromal cells has been shown to impair both decidualization and its interaction with trophoblast cells (Deryabin and Borodkina 2022). In contrast, Woods et al. experimentally demonstrated that transplanting embryos from aged mice into a young uterine environment can improve embryo implantation rates (Woods et al. 2017). These findings suggested that ameliorating the senescent microenvironment in the endometrium may help resolve decidualization defects and promote embryo implantation. However, the molecular mechanism underlying age‐related decidualization defects remains poorly understood (Pathare et al. 2023). Elucidating these mechanisms may facilitate early identification and targeted interventions for age‐related infertility.

RICTOR, a core subunit of the mTORC2 complex, has been implicated in various age‐associated pathologies. Hepatic *Rictor* deficiency in mice significantly shortens male lifespan (Lamming et al. 2014), and recent evidence indicates a role for RICTOR in endometrial epithelial function during implantation (Zhang et al. 2021). Nevertheless, the impact of RICTOR on stromal cells and decidualization remains unclear. Whether RICTOR dysfunction contributes to decidualization failure in AMA women is therefore not yet understood.

The Akt3–FoxO1 signaling axis is critical for decidualization (Adiguzel and Celik‐Ozenci 2021; Peng et al. 2021; Wang et al. 2021). Akt3 functions as a central signaling node by phosphorylating FoxO1, which induces its nucleo‐cytoplasmic shuttling and relieves transcriptional repression of key decidual markers such as *PRL* and *IGFBP1* (Adiguzel and Celik‐Ozenci 2021; Wang et al. 2021). Given that RICTOR is necessary for Akt phosphorylation at Ser473, an essential step for FoxO1 nuclear exclusion (Liu et al. 2020; Sarbassov et al. 2005), we hypothesize that RICTOR regulates decidualization through Akt3–FoxO1 signaling, and that dysregulation of this pathway may contribute to age‐related decidualization failure.

In this study, we aimed to investigate the role of RICTOR in AMA‐associated decidualization defects and to delineate its mechanistic involvement in the Akt3–FoxO1 pathway. Our findings may provide new diagnostic and therapeutic strategies for age‐related infertility.

## 2 Methods

### 2.1 Decidual Samples Collection

This study collected decidual specimens from two groups: one consisting of AMA women who underwent induced abortion due to inevitable miscarriage, and the other comprising young women who elected termination for social reasons. The groups were defined as follows: (a) Aged group: women aged 35–45 years who underwent first‐trimester spontaneous abortion (gestational age < 12 weeks) due to inevitable miscarriage; (b) Young group: women aged 20–30 years who underwent first‐trimester induced abortion (gestational age < 12 weeks) for social reasons. Women aged 31–34 years were excluded to minimize age overlap and maintain a clear distinction between groups. All participants included in the study had chromosomally normal embryos. Exclusion criteria were as follows: (a) history of hormone‐based miscarriage prevention therapy; (b) known diagnosis or carrier status of a monogenic genetic disorder, or presence of chromosomal abnormalities; (c) other diseases that may affect female reproductive health.

### 2.2 Isolation and Identification of hESCs

Endometrial tissues were collected from young (*n* = 20) and AMA (*n* = 20) women with regular menstrual cycles who underwent hysteroscopic surgery at the Women's Hospital of Zhejiang University School of Medicine. All participants underwent hysteroscopy and endometrial biopsy as part of an infertility evaluation and had not received hormone therapy for at least 3 months prior to surgery. Proliferative‐phase endometrial samples were collected aseptically for the isolation of primary human endometrial stromal cells (hESCs), following a previously described protocol (Lin et al. 2022). The isolated cells from both groups were cultured in DMEM/F12 medium supplemented with 10% fetal bovine serum (FBS) and 1% penicillin–streptomycin at 37°C in a humidified atmosphere containing 5% CO_2_. The culture medium was refreshed every 2 to 3 days, and cells were passaged at a ratio of 1:3 to 1:4 prior to cryopreservation.

### 2.3 In Vitro Decidualization Induction

To induce decidualization, hESCs were treated for 4 days (designated as YD) with a well‐established hormonal cocktail containing medroxyprogesterone acetate (MPA, 1 × 10^−6^ M; Sigma‐Aldrich, M1629), cyclic adenosine monophosphate (cAMP, 5 × 10^−5^ M; Sigma‐Aldrich, D0627), and β‐estradiol (E₂, 1 × 10^−8^ M; Sigma‐Aldrich, 50–28‐2) in phenol red‐free DMEM/F12 medium containing 2% charcoal‐stripped FBS. Successful decidualization was confirmed by: (a) morphological assessment of actin cytoskeleton reorganization using Actin‐Tracker Red‐594 staining, performed according to the manufacturer's instructions; and (b) quantification of classic decidual markers (*PRL* and *IGFBP1*) via RT‐qPCR.

### 2.4 siRNA Transfection

Cells were seeded in 6‐well plates and allowed to reach 30% confluence prior to transfection. The transfection complex was prepared by gently mixing 100 nM siRNA with 5 μL jetPRIME transfection reagent in 200 μL jetPRIME buffer, followed by incubation at room temperature for 10 min. Before transfection, cells were washed with PBS, and the medium was replaced with 1 mL culture medium. The prepared transfection mixture (200 μL per well) was then added dropwise to the cell cultures. Cells were maintained at 37°C in a 5% CO_2_ environment. After 24/48 h of incubation, cells were harvested for subsequent analysis. Rictor siRNA sequences: Sense: 5′‐GCGGUUAGCUUUAUUAAAUTT‐3′. Antisense: 3′‐AUUUAAUAAAGCUAACCGCTT‐5′. Scrambled control siRNA sequences: Sense: 5′‐TTCGGTCCAGTTGCCTTCTCC‐3′. Antisense: 3′‐TCTGAAGAGGTGAGTGGCTGTC‐5′.

### 2.5 shRNA And Lentiviral Transduction

Lentiviruses carrying shRNA targeting human RICTOR and nonspecific negative control NC lentivirus were purchased from Genepharma (Shanghai, China). Target shRNA sequences for human RICTOR were: shRNA‐1: 5′‐GCGGTTAGCTTTATTAAAT‐3′; shRNA‐2: 5′‐GAGCATGCATTGCCATTAT‐3′. The sequence of negative control NC shRNA was 5′‐TTCTCCGAACGTGTCACGT‐3′. All shRNA cassettes were inserted into the LV3 (H1/GFP&Puro) lentiviral vector, and the identity of all constructed lentiviral plasmids was verified by DNA sequencing. hESCs were seeded in 24‐well plates and cultured overnight at 37°C, followed by transduction with 1:100 diluted lentivirus in DMEM/F12 (500 μL/well) containing 5 μg/mL Polybrene. Medium was refreshed 24 h later, and cells were incubated for another 2 days (3 days total post‐transduction). Fluorescence was assessed at 72 h before puromycin selection for stable knockdown cell lines.

### 2.6 Immunofluorescence Staining (IF) of hESCs

Treated hESCs were fixed with 4% paraformaldehyde for 15 min, permeabilized with 0.5% Triton X‐100 for 30 min at room temperature, and blocked with 5% BSA for 30 min at room temperature. The cells were then incubated overnight at 4°C with an anti‐FoxO1 primary antibody (Cell Signaling Technology, #2880). After washing, the cells were stained with a goat anti‐rabbit secondary antibody at 37°C for 30 min. Nuclei were counterstained with DAPI. Images were captured using an Olympus IX81‐FV100 microscope.

### 2.7 Extraction of RNA for RT‐qPCR

Total RNA was extracted from hESCs using Trizol reagent (Invitrogen, USA) according to the manufacturer's protocol. Then, 1 μg of total RNA was reverse‐transcribed into cDNA using the MMLV Reverse Transcriptase cDNA Synthesis Kit (Vazyme, China). Quantitative real‐time PCR (qPCR) was performed on a Roche LightCycler 480II system. The primer sequences used for qPCR are listed in Table S1. Gene expression levels were normalized to GAPDH and quantified using the comparative threshold cycle (2^⁻⁻^ΔΔCT^) method. All samples were analyzed in three or four technical replicates.

### 2.8 Protein Isolation and Western Blot Analysis

Both human and mouse tissues were lysed in RIPA lysis buffer (CST, #9806) supplemented with protease and phosphatase inhibitor cocktails (TargetMol, #C0001, #C0002, #C0003), as previously described (Lin et al. 2022). Protein concentrations were determined using a bicinchoninic acid (BCA) assay kit (Thermo Scientific, #23227). Equal amounts of protein were denatured in 5× SDS‐PAGE loading buffer (CWBIO, #CW0028S) by heating, separated by SDS‐PAGE, and subsequently transferred onto nitrocellulose membranes. After blocking with 5% BSA, the membranes were incubated overnight at 4°C with the following primary antibodies: mTOR (1:1000; #ET1608‐5; HuaBio), p‐mTOR (Ser2448) (1:1000; #67778–1‐Ig; Proteintech), RICTOR (1:1000; #HA721142; HuaBio), RAPTOR (1:1000; #20984–1‐AP; Proteintech), AKT3 (1:1000; #3788; CST), p‐AKT3 (Ser473) (1:1000; #4060; CST), FoxO1 (1:1000; #2880; CST), PI3K (1:1000; #4249; CST), Lamin B1 (1:20000; #ET1606‐27; HuaBio), GAPDH (1:50000; #60004–1‐Ig; Proteintech), and β‐actin (1:20000; #66009–1‐Ig; Proteintech). Following washes, the membranes were incubated with HRP‐conjugated goat anti‐rabbit IgG (1:5000; #BK‐R050; Bioker) or goat anti‐mouse IgG (1:5000; #BK‐M050; Bioker) for 1 h at room temperature. Protein bands were visualized using an ECL detection reagent and imaged with a chemiluminescence imaging system (Protein Simple, Vilber GmbH, France). Band intensities were quantified using Image J software.

### 2.9 In Vivo Decidualization Induction

Young (8‐week‐old) and aged (10‐month‐old) female ICR mice were housed under a 12‐h light/dark cycle with ad libitum access to food and water. To establish induced in vivo decidualization, these mice underwent ovariectomy and were allowed to recover for 14 days. Subsequently, they were subcutaneously injected with 100 ng of estradiol (E2; Sigma‐Aldrich, #E2758) daily for three consecutive days, followed by a 2‐day rest period. Starting on day 20 (designated as 1 dpc), mice received daily subcutaneous injections of 1 mg of progesterone (P4; Sigma‐Aldrich, #P0130) and 10 ng of E2 for 8 days. On 4 dpc, intrauterine injection of 20 μL of sesame oil was administered into one uterine horn to induce decidualization (coded as Oil), while the contralateral horn was injected with normal saline as control (coded as Ctr). Decidual tissues were harvested at 8 dpc for subsequent analysis.

### 2.10 Embryo Transfer

Blastocysts were collected from 8‐week‐old donor ICR mice mated with fertile males (vaginal plug = E1) and flushed from the uterine horns on E4 using M2 medium. Recipient mice were hormonally synchronized (eCG/hCG = 10 IU) and mated with vasectomized males to induce pseudopregnancy (vaginal plug = 1 dpc). On E4, 16 blastocysts (8 per horn) were surgically transferred to the 3 dpc pseudopregnancy mice. Decidual tissues were harvested on 11 dpc for further experiments.

### 2.11 MHY1485 Treatment in Aged Mice

MHY1485 (Med Chem Express, #HY‐B0795) was prepared as a 10 mM stock solution and stored at −80°C. For in vivo administration, the stock solution was diluted in corn oil to a final volume of 200 μL (containing 70 μL of stock and 130 μL corn oil). Aged ICR mice received daily intraperitoneal injections of this solution for three consecutive days prior to embryo transfer or decidualization induction. Control mice were injected with an equivalent volume of corn oil vehicle on the same schedule. Following this pretreatment, the mice underwent either embryo transfer using blastocysts from young donors or artificial decidualization as described in the previous sections.

### 2.12 RNA‐Sequencing (RNA‐Seq)

Total RNA was extracted from uterine tissues of control (*Rictor* ^f/f^) and Rictor cKO (*Rictor* ^d/d^) mice (*n* = 4 per group). Sequencing libraries were constructed from 1 μg of total RNA per sample. Following total RNA extraction, poly (A) + mRNA libraries were prepared and sequenced on an Illumina platform (2 × 150 bp). After quality control, the reads were aligned to the mm10 genome. Differential expression analysis identified genes with a fold change > 2 and an adjusted *p*‐value < 0.05. These significant DEGs were subjected to GO and KEGG enrichment analyses. The raw RNA‐seq data have been deposited in the NCBI Gene Expression Omnibus under the accession number GSE310105, which is currently private and will be released on 2029‐11‐16.

### 2.13 Histological Analysis and Immunohistochemistry

Uterine tissues were fixed in 4% PFA, paraffin‐embedded, and cut into 3–4 μm sections for H&E staining and immunohistochemistry (IHC). H&E staining was performed using standard methods (Lin et al. 2022), and slides were digitized with an Olympus VS200 scanner. For IHC, sections were immunostained with an anti‐RICTOR primary antibody (1:200; HuaBio, #HA721142) and a corresponding secondary antibody (DakoCytomation, #GK600711). RICTOR expression was semi‐quantified by two blinded observers applying the immune response score (IRS) system (Lin et al. 2022). Image analysis was conducted using Image Pro‐Plus 6.0.

### 2.14 Statistical Analysis

Statistical analyses were performed using GraphPad Prism 8.0. Data were presented as the mean ± SEM. Comparisons between two independent groups were performed using an unpaired two‐tailed Student's *t*‐test. Comparisons among three or more groups involving a single experimental factor were performed using ordinary one‐way ANOVA followed by Dunnett's multiple‐comparisons test when multiple experimental groups were compared with a single control group or Tukey's multiple‐comparisons test when all pairwise comparisons were performed. Experiments involving two independent factors were analyzed using two‐way ANOVA followed by Šídák's multiple‐comparisons test for prespecified comparisons. All statistical tests were two‐sided, and *p* value < 0.05 was considered statistically significant. Each experiment included at least three independent biological replicates. The exact sample size and statistical test used were indicated in the corresponding figure legends.

## 3 Results

### 3.1 Impaired Decidualization in Aged Females

**FIGURE 1:**
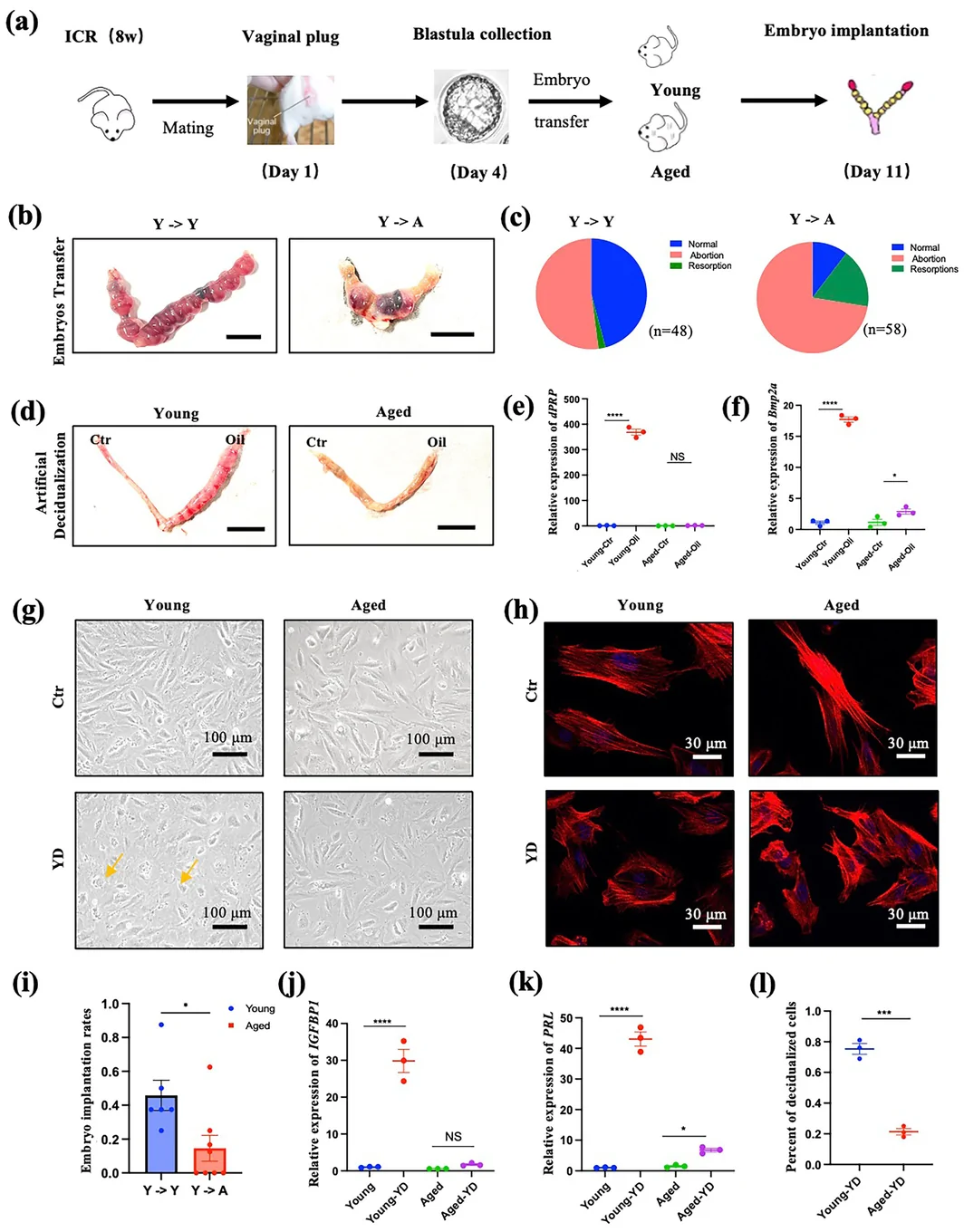
Impaired decidualization in aged females. (a) Schematic of the embryo transfer experiment. (b) Representative images of uteri from young (Y‐ > Y) and aged (Y‐ > A) recipients at gestational day 11. Scale bar = 2 cm. (c) Pie charts showing the proportion of normal, abnormal, and resorbed embryos. (d) Uterine morphology after artificial decidualization induction in young and aged mice. Scale bar = 2 cm. (e, f) mRNA levels of decidual markers *dPRP* and *Bmp2a* in uterine tissues from young and aged mice. Oil denotes induced decidualization, and Ctr denotes the untreated control, respectively. Data represent mean ± SEM, NS means *p* > 0.05, * *p* < 0.05, ****p < 0.0001, *n* = 3. Statistical significance was assessed using two‐way ANOVA followed by Šídák's multiple‐comparisons test. (g) Phase‐contrast images of young and aged hESCs after 4 days of decidual induction. YD denotes induced decidualization, and Ctr denotes the untreated control, respectively. Differentiated cells exhibiting a rounded morphology are indicated by arrows. Scale bar = 100 μm. (h) Representative IF staining of filamentous Actin (red) and nuclear marker DAPI (blue) in young and aged hESCs. YD denotes induced decidualization, and Ctr denotes the untreated control, respectively. Scale bar = 30 μm. (i) Embryo implantation rates in “Y‐ > Y” and “Y‐ > A” groups at gestational day 11. Data were presented as mean ± SEM. *p < 0.05, *n* = 6. Statistical significance was assessed using an unpaired two‐tailed Student's *t*‐test. (j, k) mRNA expression of decidual markers *IGFBP1* and *PRL* in young and aged hESCs after in vitro decidualization. Data were presented as mean ± SEM. NS means *p* > 0.05, *p < 0.05, ****p < 0.0001, *n* = 3. Statistical significance was assessed using two‐way ANOVA followed by Šídák's multiple‐comparisons test. (l) Quantitative analysis of hESC decidualization efficiency. Data were presented as mean ± SEM. ***p < 0.001, *n* = 3. Statistical significance was assessed using an unpaired two‐tailed Student's *t*‐test.

To isolate the contribution of uterine factors from embryonic influences on adverse pregnancy outcomes in AMA mice, we established an embryo transfer model. As illustrated in Figure 1a, blastocysts from young donors were transferred into the uteri of either young (**Y‐ > Y**) or aged (**Y‐ > A**) pseudopregnant recipients. Assessment at day 7 post‐transfer revealed that aged recipients had significantly lower implantation rates and decidualization levels, alongside higher rates of miscarriage and embryo resorption compared to young recipients (Figure 1b,c,i).

We next specifically evaluated the uterine decidualization capacity using an artificial induction assay. In young mice, intrauterine injection of sesame oil triggered a robust decidual response, characterized by pronounced endometrial thickening and vascular congestion in the stimulated horn. This response was markedly blunted in aged mice (Figure 1d). Consistently, oil stimulation strongly induced the expression of the decidual markers *dPRP* and *Bmp2a* in young mice. In aged mice, oil stimulation did not significantly increase *dPRP* expression but induced a modest increase in *Bmp2a* expression. Nevertheless, the overall induction of these decidual markers was substantially attenuated in aged mice (Figure 1e,f).

To determine whether this defect is conserved in humans, we isolated hESCs from young and aged women and induced decidualization in vitro. Following induction, hESCs from young donors underwent characteristic transformation, evident as cellular enlargement, rounding, and multinucleation (Figure 1g,h,l), and showed significant upregulation of the decidual markers *IGFBP1* and *PRL* (Figure 1j,k). In contrast, hESCs from aged women exhibited markedly impaired morphological decidualization. Decidualization induction did not significantly increase *IGFBP1* expression in aged hESCs, although a modest but statistically significant increase in *PRL* expression was observed. The magnitude of this molecular response remained substantially lower than that observed in young hESCs.

Collectively, these results from mouse models and human primary cells demonstrated that AMA is associated with an intrinsic uterine defect, characterized by impaired decidualization and compromised embryo implantation.

### 3.2 Reduced RICTOR Expression in the Decidua of AMA Women

**FIGURE 2:**
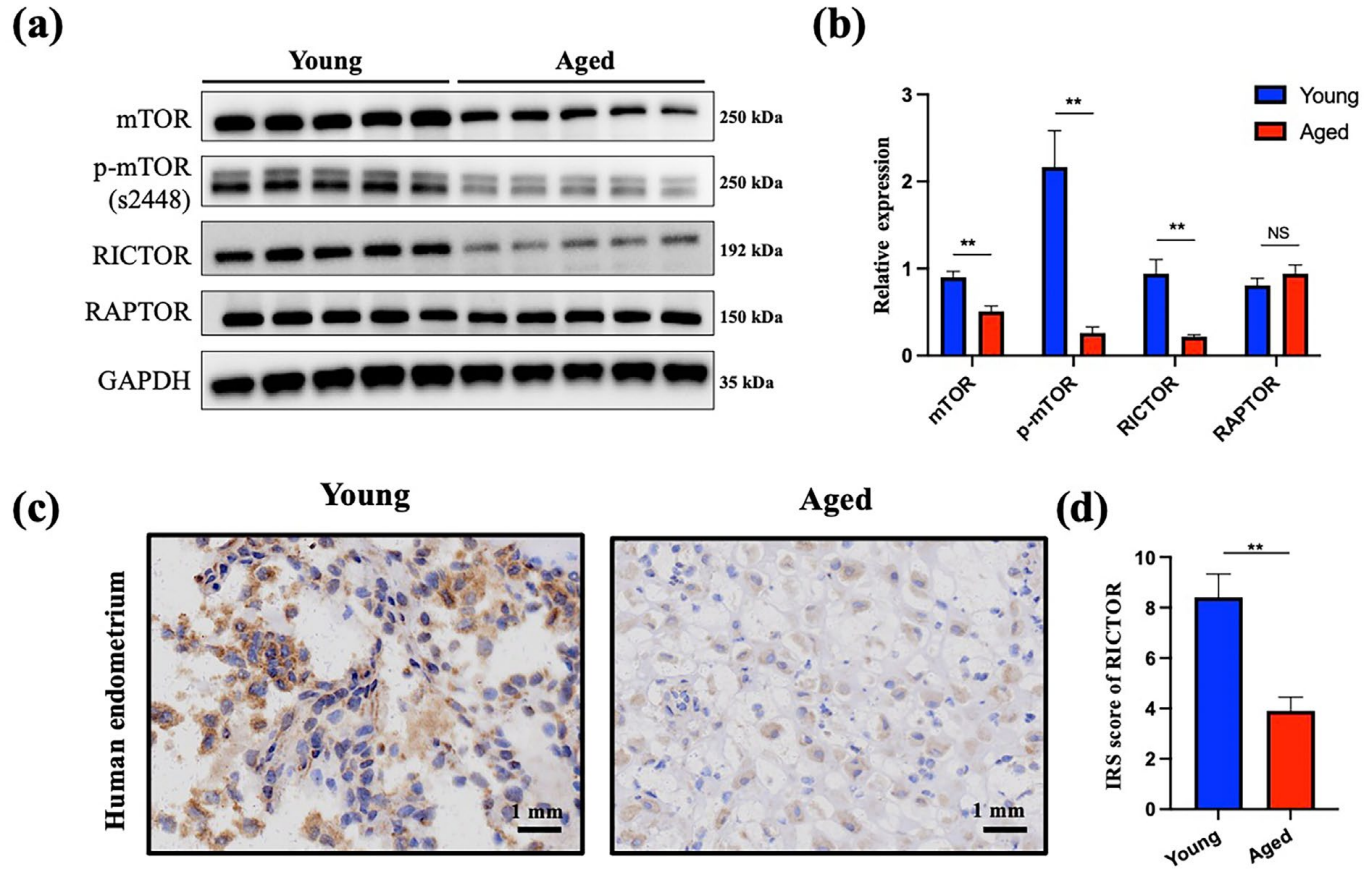
Reduced RICTOR expression in the decidua of AMA women. (a) Representative Western blots of mTOR, p‐mTOR, RICTOR, and RAPTOR protein levels in decidual tissues from young and AMA women. GAPDH was used as a loading control. (b) Quantitative analysis of protein levels from (a). Data were normalized to GAPDH and are presented as mean ± SEM. NS means *p* > 0.05, **p < 0.01, ***p < 0.001, ****p < 0.0001, *n* = 5. Statistical significance was assessed using an unpaired two‐tailed Student's *t*‐test. (c) Representative IHC images showing RICTOR staining in decidual tissues. Scale bar = 1 mm. (d) Quantitative analysis of RICTOR expression based on the IRS from IHC staining. Data were presented as mean ± SEM. **p < 0.01, *n* = 5. Statistical significance was assessed using an unpaired two‐tailed Student's *t*‐test.

We analyzed the expression of mTOR, p‐mTOR, RAPTOR (a key component of mTORC1), and RICTOR (a key component of mTORC2) in the decidua of young and aged women. The results revealed a significant decrease in RICTOR protein levels in the decidua of AMA women compared to young controls, whereas RAPTOR expression remained unchanged (Figure 2a,b). IHC analysis further demonstrated that RICTOR was predominantly localized in decidual stromal cells and was markedly downregulated in the AMA group (Figure 2c,d).

### 3.3 RICTOR Deficiency Impaired in Vitro Decidualization in hESCs

**FIGURE 3:**
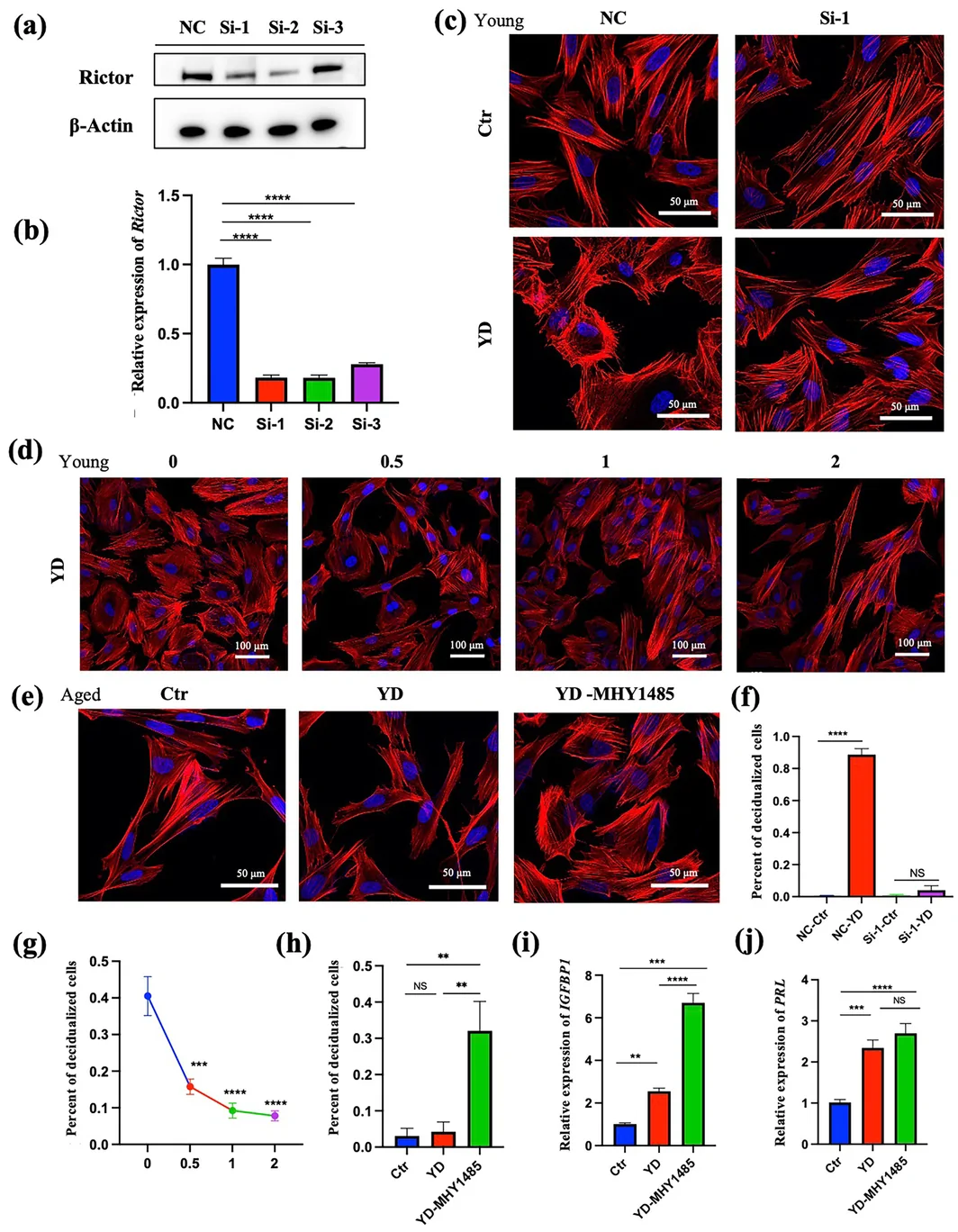
RICTOR deficiency impaired in vitro decidualization in hESCs. (a) Representative Western blots showing RICTOR protein expression in hESCs from young women after transfection with control (NC) or RICTOR‐targeting siRNAs (Si‐1, Si‐2, Si‐3). (b) Quantification of RICTOR protein levels from (a), normalized to β‐Actin. Data were represented as mean ± SEM, ****p < 0.0001, *n* = 3. Statistical significance was assessed using ordinary one‐way ANOVA followed by Dunnett's multiple‐comparisons test, with the NC group serving as the respective controls (c) Representative IF images of hESCs after 4 days in vitro decidualization induction. Cells were transfected with NC or Si‐1 siRNA and stained for F‐Actin (phalloidin, red) and nuclei (DAPI, blue). YD denotes induced decidualization, and Ctr denotes the untreated control, respectively. (d) Representative images of hESCs from young women treated with the RICTOR inhibitor JR‐AB2‐011 (0, 0.5, 1, 2 μM) during decidualization induction, visualized by F‐Actin/DAPI staining. (e) Representative images of hESCs from AMA women treated with or without the RICTOR agonist MHY1485 (10 μM) during decidualization induction, visualized by F‐Actin/DAPI staining. (f) Quantitative analysis of the percentage of decidualized cells in (c). Data were presented as mean ± SEM. NS means *p* > 0.05, ****p < 0.0001, *n* = 6. Statistical significance was assessed using two‐way ANOVA followed by Šídák's multiple‐comparisons test. (g) Quantitative analysis of the percentage of decidualized cells in (d). Data were presented as mean ± SEM. ****p < 0.0001, ***p < 0.001, *n* = 5. Statistical significance was assessed using ordinary one‐way ANOVA followed by Dunnett's multiple‐comparisons test, with the 0 μM group serving as the respective controls (h) Quantitative analysis of the percentage of decidualized cells in (e). Data were presented as mean ± SEM. NS means *p* > 0.05, **p < 0.01, *n* = 6. Statistical significance was assessed using ordinary one‐way ANOVA followed by Tukey's multiple‐comparisons test. (i, j) mRNA expression levels of decidual markers *IGFBP1 and PRL* in hESCs from AMA women treated with or without MHY1485. Data were presented as mean ± SEM. NS means *p* > 0.05, **p < 0.01, ***p < 0.001, ****p < 0.0001, *n* = 6. Statistical significance was assessed using ordinary one‐way ANOVA followed by Tukey's multiple‐comparisons test.

To investigate the functional role of RICTOR in decidualization, we first knocked down endogenous RICTOR in hESCs via transient siRNA transfection. Given its higher knockdown efficiency, Si‐1 was chosen for subsequent functional assays (Figure 3a,b). Following in vitro decidualization induction for 4 days, phalloidin staining revealed that RICTOR knockdown by Si‐1 markedly impaired the characteristic morphological changes associated with decidualization compared to the negative control (NC) group (Figure 3c,f).

To further validate our observations and exclude off‐target effects from transient siRNA, we constructed two stable RICTOR‐knockdown cell lines using lentiviral shRNAs designed based on the sequences of Si‐1 and Si‐2 (coded as shRNA‐1 and shRNA‐2). Successful stable knockdown was verified in Figure S1. Consistent with our siRNA data, stable RICTOR deficiency significantly suppressed both decidual morphological changes and the mRNA expression of canonical decidual markers *IGFBP1* and *PRL* after 4 days of decidual induction (Figure S2).

To further corroborate these findings, we treated hESCs from young women with JR‐AB2‐011, a specific RICTOR inhibitor. Decidualization capacity was progressively diminished with increasing inhibitor concentrations (Figure 3d,g). Conversely, treatment with the RICTOR agonist MHY1485 in hESCs from AMA women significantly rescued the decidualization defect, as evidenced by an increased proportion of cells exhibiting decidual morphology (Figure 3e,h) and by elevated expression levels of the decidual markers *IGFBP1* and *PRL* (Figure 3i,j).

Collectively, these results demonstrated that RICTOR is essential for proper decidualization.

### 3.4 Uterine‐Specific Ablation of *Rictor* Impaired Decidualization Independent of Embryonic Factors

While previous studies have linked Rictor deficiency to defective embryo implantation (Zhang et al. 2021), it was unknown whether this phenotype was primarily due to an intrinsic uterine defect or to extrinsic embryonic signals, since murine decidualization is typically initiated by embryonic stimulation.

**FIGURE 4:**
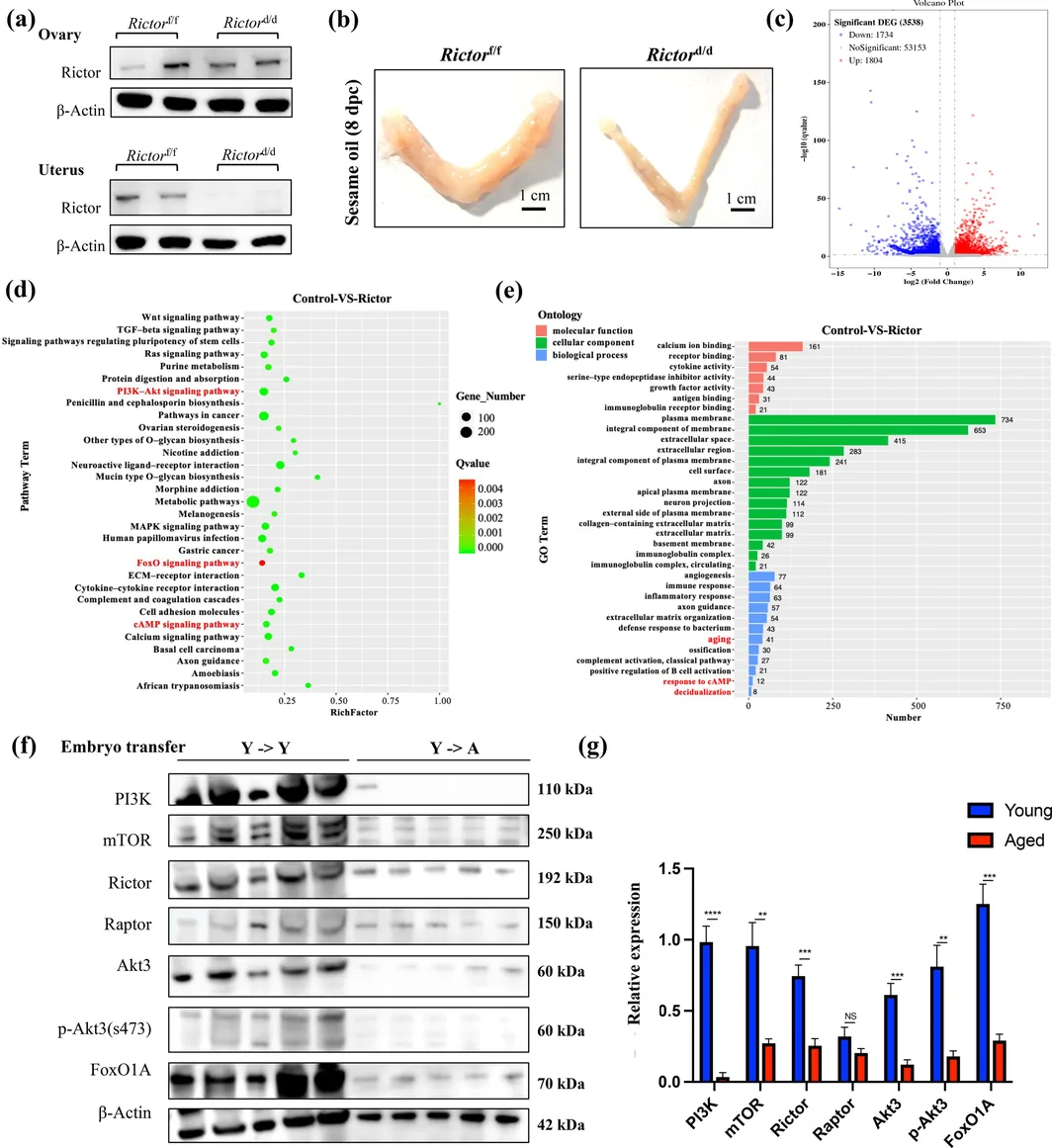
Uterine‐specific ablation of *Rictor* impaired decidualization independent of embryonic factor. (a) Western blot analysis of Rictor protein expression in uterine and ovarian tissues from *Rictor* cKO (*Rictor* ^d/d^) and control mice (*Rictor* ^f/f^). β‐Actin was used as a loading control. (b) Representative images of uterine morphology following artificial decidualization induction in *Rictor* ^f/f^ and *Rictor* ^d/d^ mice, *n* = 4 (c) Volcano plot displaying differentially expressed genes in E8 decidual tissues from *Rictor* ^f/f^ and *Rictor* ^d/d^ mice, *n* = 4. (d, e) Gene Ontology (GO) and KEGG pathway enrichment analyses of differentially expressed genes from (c). (f) Western blot analysis of PI3K, mTOR, Rictor, Raptor, Akt3, p‐Akt3 (Ser473), and FoxO1 protein levels in decidual tissues from the Y‐ > Y and Y‐ > A embryo transfer groups. β‐Actin was used as a loading control, *n* = 5. (g) Quantification of protein expression levels was shown in (f). Data are presented as mean ± SEM, NS means *p* > 0.05, * *p* < 0.05, **p < 0.01, ***p < 0.001, ****p < 0.0001, *n* = 5. Statistical significance was assessed using an unpaired two‐tailed Student's *t*‐test.

To specifically assess the intrinsic role of Rictor in the uterine decidual response, we generated uterine‐specific *Rictor* conditional knockout (*Rictor* ^d/d^) mice, which showed a complete loss of Rictor protein compared to controls (*Rictor* ^f/f^) (Figure 4a). We then induced artificial decidualization by sesame oil stimulation to exclude embryonic factors. Notably, *Rictor* ^d/d^ mice failed to undergo decidualization, whereas control mice exhibited robust uterine swelling (Figure 4b). RNA‐seq analysis of decidual tissues revealed 1804 upregulated and 1734 downregulated genes in *Rictor* ^d/d^ mice compared to controls (Figure 4c). KEGG pathway analysis showed significant enrichment in key processes, including the FoxO, PI3K‐Akt, and cAMP signaling pathways, as well as GO biological terms enriched in the aging, response to cAMP, and decidualization processes (Figure 4d,e). Consistent with transcriptomic results, Raptor protein expression showed no significant differences, while PI3K, Akt3, Rictor, and FoxO1 were downregulated in decidual tissues of Y‐ > A mice following embryo transfer (Figure 4f,g).

### 3.5 RICTOR Deficiency Impaired Nuclear Export of FoxO1 in hESCs

Our RNA‐seq pathway analysis identified the FoxO signaling pathway as one of the most significantly altered pathways upon Rictor ablation. Since the nucleo‐cytoplasmic shuttling of FoxO1 is critical for proper decidualization (Adiguzel and Celik‐Ozenci 2021), we hypothesized that RICTOR might regulate this process. While FoxO1 promotes the transcription of decidual markers (e.g., *IGFBP1* and *PRL*) in the nucleus, its physiological nuclear accumulation toward the end of decidualization contributes to program termination (Adiguzel and Celik‐Ozenci 2021). Conversely, impaired nuclear export is known to disrupt the decidualization process (Adiguzel and Celik‐Ozenci 2021).

**FIGURE 5:**
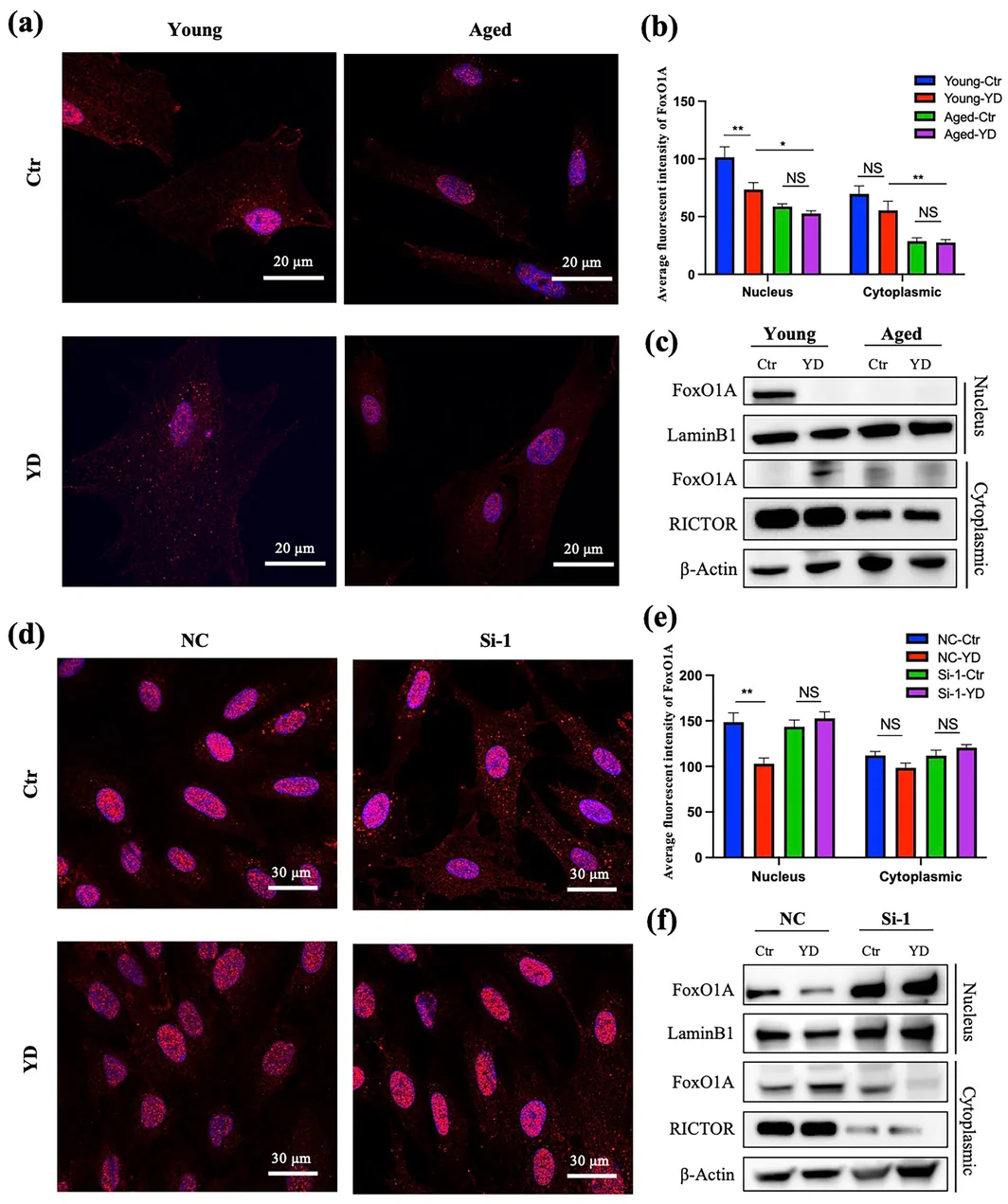
RICTOR deficiency impaired nuclear export of FoxO1 in hESCs. (a) Representative IF images of FoxO1 (red) in primary hESCs from young and aged women, with or without in vitro decidualization induction. Nuclei are counterstained with DAPI (blue). YD denotes induced decidualization, and Ctr denotes the untreated control, respectively. (b) Quantification of FoxO1 fluorescence intensity from (a). Data are presented as mean ± SEM, NS means *p* > 0.05, * *p* < 0.05, **p < 0.01, *n* = 4. Statistical significance was assessed using two‐way ANOVA followed by Šídák's multiple‐comparisons test. (c) Subcellular fractionation showing FoxO1 subcellular localization and cytoplasmic RICTOR levels in young and aged hESCs. Lamin B1 (nuclear) and β‐Actin (cytoplasmic) served as loading controls. *n* = 3. (d) IF staining of FoxO1 (red) in control (NC) and *RICTOR*‐knockdown (Si‐1) young hESCs, with or without decidualization. (e) Quantification of FoxO1 intensity from (d). Data are presented as mean ± SEM, NS means *p* > 0.05, **p < 0.01, *n* = 5. Statistical significance was assessed using two‐way ANOVA followed by Šídák's multiple‐comparisons test. (f) Subcellular fractionation western blot analysis of FoxO1 localization and cytoplasmic RICTOR expression in NC and Si‐1young hESCs. Lamin B1 and β‐Actin served as nuclear and cytoplasmic loading controls, respectively. *n* = 3.

Consistent with our hypothesis, IF and Western blot analyses revealed substantial nuclear accumulation of FoxO1 in aged hESCs, which indicated defective nuclear export (Figure 5a–c). We next explored whether this defect was directly caused by RICTOR deficiency. To test this, we knocked down RICTOR in young hESCs (Si‐1 group) and observed the cytoplasmic/nuclear FoxO1 ratio was markedly decreased, accompanied by evident FoxO1 nuclear retention (Figure 5d–f, Figure S3), phenocopying the aged cells.

### 3.6 RICTOR Activation Rescued Age‐Related Decidualization Defects in Vivo

**FIGURE 6:**
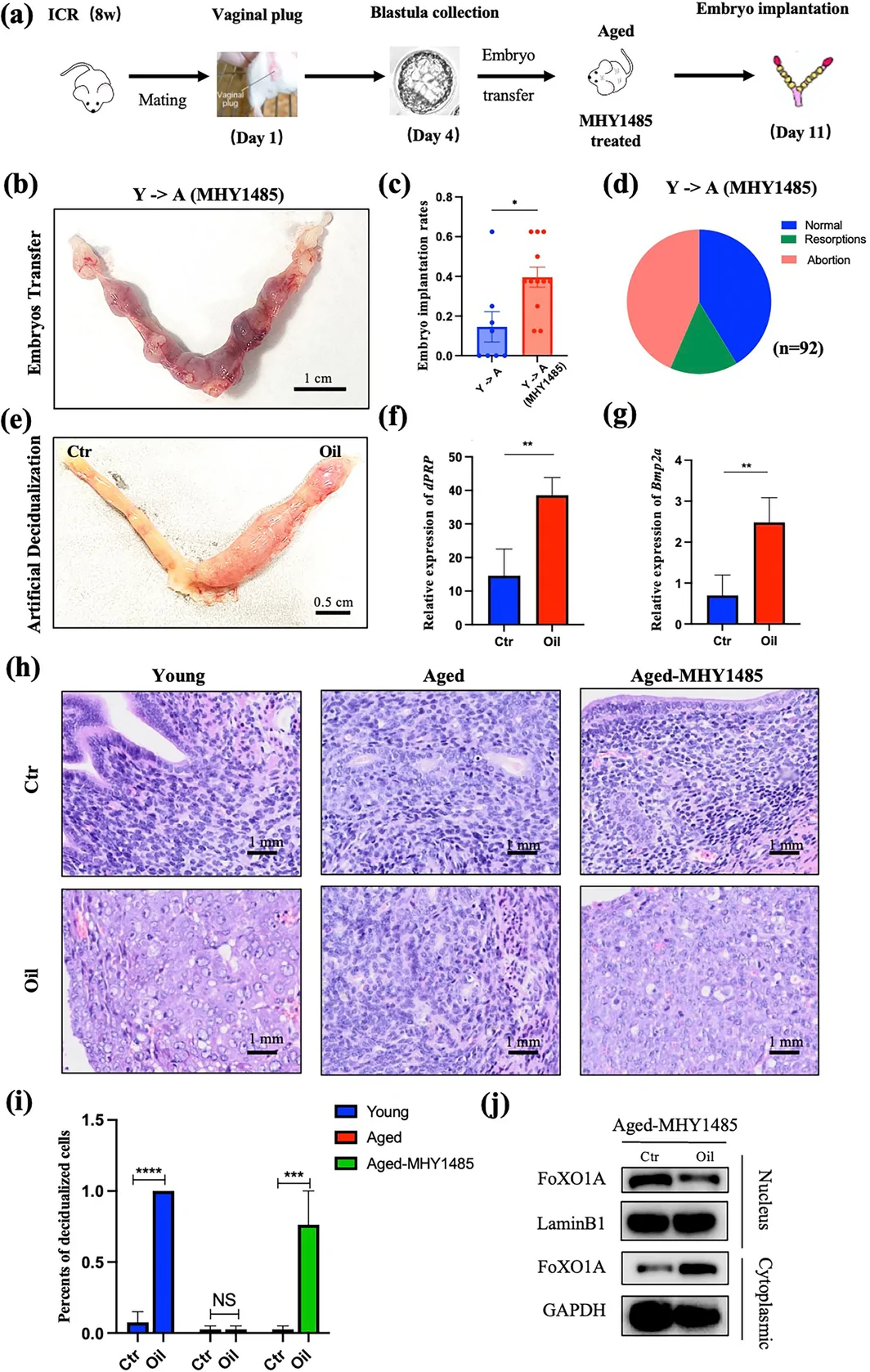
RICTOR activation rescued age‐related decidualization defects in vivo. (a) Schematic of the embryo transfer experiment in aged mice pretreated with MHY1485 or vehicle. (b) Representative images of uteri from Y‐ > A treated with MHY1485 at gestational day 11. (c) Embryo implantation rates in aged recipients. Data were presented as mean ± SEM. *p < 0.05. Statistical significance was assessed using an unpaired two‐tailed Student's *t*‐test. (d) The distribution of embryo phenotypes (normal, abnormal, resorbed) was shown as pie charts. (e) Uterine morphology following artificial decidualization in aged mice with or without MHY1485 treatment, *n* = 4. (f, g) mRNA expression levels of decidual markers *dPRP* and *Bmp2a* in MHY1485‐treated aged uteri. Data represent mean ± SEM. *p < 0.01, *n* = 4. Statistical significance was assessed using an unpaired two‐tailed Student's *t*‐test. (h) H&E staining of uterine sections from saline‐perfused (top) and oil‐induced decidualized (bottom) uteri of young, aged, and MHY1485‐treated aged mice, *n* = 4. (i) Quantification of decidualized percents from H&E‐stained sections in (h). Data represent mean ± SEM. NS means *p* > 0.05, ***p < 0.001, ****p < 0.0001, *n* = 4. Statistical significance was assessed using two‐way ANOVA followed by Šídák's multiple‐comparisons test. (j) Subcellular fractionation analysis of FoxO1 localization by western blot in aged‐MHY1485 decidua. Lamin B1 and β‐Actin served as nuclear and cytoplasmic loading controls, respectively.

To determine whether pharmacological activation of RICTOR could ameliorate endometrial decidualization and embryo implantation defects in aged mice, we treated aged mice with the RICTOR activator MHY1485. Treated mice were allocated to two experimental paradigms: embryo transfer following mating with vasectomized males to assess implantation competence, or artificial decidualization induced by intrauterine oil infusion. MHY1485 treatment significantly improved both embryo implantation rates and decidualization levels compared with untreated aged controls (Figure 6a–e). Furthermore, the expression of decidual markers *dPRP* and *Bmp2*a was elevated in the uteri of MHY1485‐treated aged mice (Figure 6f,g), indicating functional restoration of the decidual response. Histological analysis of uterine cross sections showed a significantly higher proportion of decidualized cells in the MHY1485 group (Figure 6h,i), which was accompanied by increased FoxO1 nuclear export (Figure 6j). These results collectively demonstrate that enhancing Rictor activity is sufficient to rescue age‐associated decidualization failure and improve pregnancy outcomes in vivo.

## 4 Discussion

In summary, our study demonstrated that the downregulation of RICTOR in the decidua contributes to impaired decidualization in aged females. Using a uterine‐specific *Rictor* knockout mice model, we confirmed that *Rictor* deficiency disrupts decidualization independently of embryonic factors. Integrated transcriptomic and molecular analyses revealed a key role for the Akt3–FoxO1 pathway in this process, particularly the impaired nuclear export of FoxO1. Importantly, treatment with the Rictor activator MHY1485 significantly rescued decidualization defects and improved embryo implantation rates in aged mice.

AMA poses significant risks to reproductive success, as evidenced by increased postimplantation embryo loss and compromised fetal development (Wu et al. 2023; Zhang et al. 2022; Zhou et al. 2023). A key process implicated in these outcomes is endometrial decidualization, whose impairment can lead to implantation failure and miscarriage. To precisely define the impact of uterine aging, we first employed an embryo transfer model, which revealed that aged uteri intrinsically undermine reproductive outcomes, manifesting as reduced implantation, defective decidualizice.

AMA poses significant risks to reproductive success, as evidenced by increased postimplantation embryo loss and compromised fetal development (Wu et al. 2023; Zhang et al. 2022; Zhou et al. 2023). A key process implicated in these outcomes is endometrial decidualization, whose impairment can lead to implantation failure and miscarriage. To precisely define the impact of uterine aging, we first employed an embryo transfer model, which revealed that aged uteri intrinsically undermine reproductive outcomes, manifesting as reduced implantation, defective decidualization, and increased pregnancy loss. Furthermore, in vitro studies using hESCs from aged women confirmed cell‐autonomous defects in decidualization. Together, these findings highlight the uterus itself as a major contributor to fertility decline in AMA women.

While the mTOR signaling pathway is recognized as a key regulator of aging (Lee et al. 2024; Mannick and Lamming 2023; Mizunuma et al. 2014; Wilkinson et al. 2012), its involvement in decidua senescence remains unclear. Our data also revealed that total mTOR and phosphorylated mTOR (p‐mTOR) were notably downregulated in the decidua of aged women. mTOR functions by forming two distinct complexes, mTORC1 and mTORC2, with RAPTOR and RICTOR as their respective core components. Our results indicated that RAPTOR expression remained unaltered in AMA decidua, while RICTOR was dramatically decreased. Additionally, the IHC result also showed that RICTOR was significantly decreased in aged decidua. Given this differential expression pattern, we propose that RICTOR plays a key role in impaired decidualization in AMA women, and therefore primarily focused on exploring its function in age‐related decidualization failure. The regulatory mechanism of mTOR will be investigated in our future work. Furthermore, a study by Zhang et al. reported that Rictor/mTORC2 was involved in establishing endometrial receptivity in mice by regulating epithelial remodeling (Zhang et al. 2021). Nevertheless, the role of RICTOR in decidualization in AMA women has not been further explored. Our work showed that uterine‐specific *Rictor* knockout mice exhibited significant decidualization defects, and RNA‐seq analysis revealed enrichment of the aging, response to cAMP, and decidualization processes. Taken together, these results suggest that RICTOR acts as a critical regulator of decidualization impairment in AMA women.

FoxO1 is an important transcription factor governing endometrial stromal cell decidualization. Existing animal studies have validated that uterine‐specific *Foxo1* knockout causes obvious decidualization dysfunction (Kajihara et al. 2013), and FoxO1 is indispensable for the expression of key decidual markers *IGFBP1* and *PRL* (Adiguzel and Celik‐Ozenci 2021; Fan et al. 2012; Huang et al. 2022; Peng et al. 2021). As a downstream target of the PI3K/Akt pathway (Guo et al. 2019; Ho et al. 2025; Xiang et al. 2022), FoxO1 is phosphorylated by Akt, leading to its nuclear export and subsequent cytoplasmic retention or degradation—a process critical for decidualization to proceed (Adiguzel and Celik‐Ozenci 2021; Cheng et al. 2024; Ding et al. 2017). Consequently, defects in this export mechanism, resulting in persistent nuclear retention of FoxO1, are predicted to impair the decidualization process (Adiguzel and Celik‐Ozenci 2021). Our RNA‐seq analysis implicated the PI3K‐Akt‐FoxO axis in the decidualization disorder of uterine‐specific *Rictor* knockout mice. Consistent with this, we found that p‐Akt3 (S473) expression was downregulated in the decidua of aged mice, accompanied by a reduction in total FoxO1 protein. Furthermore, we observed a significant nuclear accumulation of FoxO1 in hESCs from aged women and in RICTOR‐knockdown hESCs from young women. This finding provides a mechanistic link between RICTOR deficiency, aberrant FoxO1 localization, and the compromised decidualization observed in AMA.

As a canonical pharmacological activator of the RICTOR pathway, MHY1485 has previously been shown to rescue perturbed retinal angiogenesis and promote cellular processes such as proliferation and wound healing (Bhakuni et al. 2024; Liu et al. 2019; Pan et al. 2017). Building on this evidence, we investigated its potential to counteract reproductive aging. We found that MHY1485 administration significantly improved embryo implantation rates and restored decidualization in aged mice. Notably, this functional restoration was associated with the effective rescue of FoxO1 nuclear export defects in the decidual stroma. Collectively, our findings underscore an essential role for RICTOR in the decidualization impairment associated with AMA and demonstrate the therapeutic potential of targeting this pathway to ameliorate age‐related fertility decline.

Therefore, RICTOR‐induced Akt3‐FoxO1 signaling is critical for the decidualization process. These results demonstrate an essential role for RICTOR in the senescent decidual and suggest that the RICTOR activator MHY1485 may represent a promising strategy to alleviate the negative effects of senescent decidua and to improve pregnancy outcomes.

In conclusion, our findings demonstrate that the RICTOR/Akt3/FoxO1 pathway is essential for proper decidualization. Loss of RICTOR in the aging uterus contributes to fertility decline. Notably, activation of RICTOR with the drug MHY1485 can effectively rescue age‐associated decidualization defects, offering a promising therapeutic strategy to improve pregnancy outcomes in AMA.

## Author Contributions

Conceptualization, R.Z., Y.W., D.Z., Y.L., D.L., Y.Y., and J.S.; Methodology, Y.L., J.S., Y.Y., D.L., and X.S.; Investigation, Y.L., D.L., and Y.Y.; Writing – original draft, Y.L.; Writing – review and editing, Y.L., J.S., X.S., D.Z., Y.W., and R.Z.; Funding acquisition, R.Z., and D.Z.; Supervision, X.S., D.Z., Y.W., and R.Z.; all authors reviewed the results and approved the final version of the manuscript.

## Funding

This work was supported by National Natural Science Foundation of China, 82394424, U23A20404. Key Research and Development Program of Zhejiang Province, 2024C03199. Natural Science Foundation of Zhejiang Province, LQN26H040005.

## Ethics Statement

All animal experiments were conducted in accordance with the national regulations on the management of experimental animals, and were approved by the experimental animal welfare ethics review committee of Zhejiang University (Approval No. ZJU20240160). Clinical studies involving human data were performed following the guidelines of the Obstetrics and Gynecology Hospital Affiliated to Zhejiang University School of Medicine (IRB‐20181015‐R), with informed consent obtained from all participants for the collection and use of biological samples.

## Conflicts of Interest

The authors declare no conflicts of interest.

## Supporting information


**Figure S1:** Validation of *RICTOR* knockdown efficiency via lentiviral shRNA. (a) Representative bright‐field and fluorescence images of human primary endometrial stromal cells (hESCs) 72 h after transfection with NC, shRNA‐1 and shRNA‐2 lentiviruses. (b) Representative Western blots showing RICTOR protein expression in hESCs from young women 72 h after transfection with NC, shRNA‐1 and shRNA‐2. (c) Quantification of RICTOR protein levels from (b), normalized to GAPDH. Data were presented as mean ± SEM, * *p* < 0.05, *n* = 3. Statistical significance was assessed using ordinary one‐way ANOVA followed by Dunnett's multiple‐comparisons test, with the NC group serving as the control. (d) mRNA expression levels of *RICTOR* in hESCs from young women 24 h after transfection with NC, shRNA‐1 and shRNA‐2. Data were presented as mean ± SEM. **** *p* < 0.0001, *n* = 4. Statistical significance was assessed using ordinary one‐way ANOVA followed by Dunnett's multiple‐comparisons test, with the NC group serving as the control.
**Figure S2:** Lentiviral‐mediated *RICTOR* knockdown impairs in vitro decidualization of hESCs. (a) Representative fluorescence images of hESCs following 4 days of decidualization induction. Cells were pre‐transduced with NC, shRNA‐1 or shRNA‐2 lentiviruses. F‐actin was labeled with phalloidin (red), and cell nuclei were counterstained with DAPI (blue). Ctr: untreated control; YD: decidualization induction group. (b) Quantitative analysis of the percentage of decidualized cells in (a). Data were presented as mean ± SEM. ns means *p* > 0.05, **** *p* < 0.0001, *n* = 5. Statistical significance was assessed using two‐way ANOVA followed by Šídák's multiple‐comparisons test. (c–d) Relative mRNA levels of decidual marker genes *IGFBP1* and *PRL* in lentivirus‐treated hESCs after 4 days of decidual induction. Data were presented as mean ± SEM. ns means *p* > 0.05, **** *p* < 0.0001, *n* = 5. Statistical significance was assessed using two‐way ANOVA followed by Šídák's multiple‐comparisons test.
**Figure S3:** Quantification of cytoplasmic/nuclear fluorescence intensity ratio of FoxO1. Data are presented as mean ± SEM, ns means *p* > 0.05, * *p* < 0.05, *n* = 4. Statistical significance was assessed using two‐way ANOVA followed by Šídák's multiple‐comparisons test.
**Table S1:** Primers used for RT‐qPCR assay.

## Data Availability

The data that support the findings of this study are available on request from the corresponding author. The data are not publicly available due to privacy or ethical restrictions.
