# Supplementary material for: RICTOR Prevents Decidualization Disorder in Women With Advanced Maternal Age via Regulating FoxO1 Nuclear Export
Source: Aging Cell. 2026 Aug 21;25(9):e70667. doi: 10.1111/acel.70667 (PMC13494740; doi:10.1111/acel.70667)
Supplement: Supplementary file 1 [file ACEL-25-e70667-s001.zip › Table S1.docx]

**Table S1. Primers used for RT-qPCR assay.**

| *dPRP-F* | GCTGCATCAATTCCTGAATGTCA |
| --- | --- |
| *dPRP-R* | CCTCATCACGTCTATACATGCG |
| *BMP2A-F* | GGGACCCGCTGTCTTCTAGT |
| *BMP2A-R* | TCAACTCAAATTCGCTGAGGAC |
| *PRL -F* | TGACCCTTCGAGACCTGTTTG |
| *PRL -R* | CTTGCTCCTTGTCTTCGGG |
| *IGFBP1 -F* | TTGGGACGCCATCAGTACCTA |
| *IGFBP1 -R* | TTGGCTAAACTCTCTACGACTCT |
| *RICTOR -F* | CCTCGATGAAGCATGTGAAGA |
| *RICTOR -R* | TCTCAGCAGGAGAAGCAAAC |
