# Supplementary material for: RICTOR Prevents Decidualization Disorder in Women With Advanced Maternal Age via Regulating FoxO1 Nuclear Export
Source: Aging Cell. 2026 Aug 21;25(9):e70667. doi: 10.1111/acel.70667 (PMC13494740; doi:10.1111/acel.70667)
Supplement: Supplementary file 1 [file ACEL-25-e70667-s001.zip › 2.0-Clean version-Supplementary-materials.pdf]

Supplementary Materials for

RICTOR prevents decidualization disorder in women with advanced maternal age via regulating FoxO1 nuclear export

Yifeng Lin[1,2,3†], Jiwei Sun[1,2†], Yue Ying[1,2†], Dan Li[1,2], Yuewen Ying[1,2], Xilin Shen[1,2], Xiao Sun[1,2], Dan Zhang[1,2*], Yiqing Wu[1,2*], Runju Zhang[1,2*]

1. Institute of Medical Genetics and Development, Key Laboratory of Reproductive Genetics (Ministry of Education) and Department of Reproductive Endocrinology, Women's Hospital, School of Medicine, Zhejiang University, Zhejiang, 310006, China.

2. Zhejiang Provincial Birth Defect Control and Prevention Research Center, Hangzhou, Zhejiang 310006, China.

3. Center for Reproductive Medicine, The First Affiliated Hospital, School of Medicine, Zhejiang University, Hangzhou 310003, Zhejiang, China.

*Corresponding author: 5311005@zju.edu.cn, wuyiqing@zju.edu.cn, zhangdan@zju.edu.cn.

† These authors contributed equally.

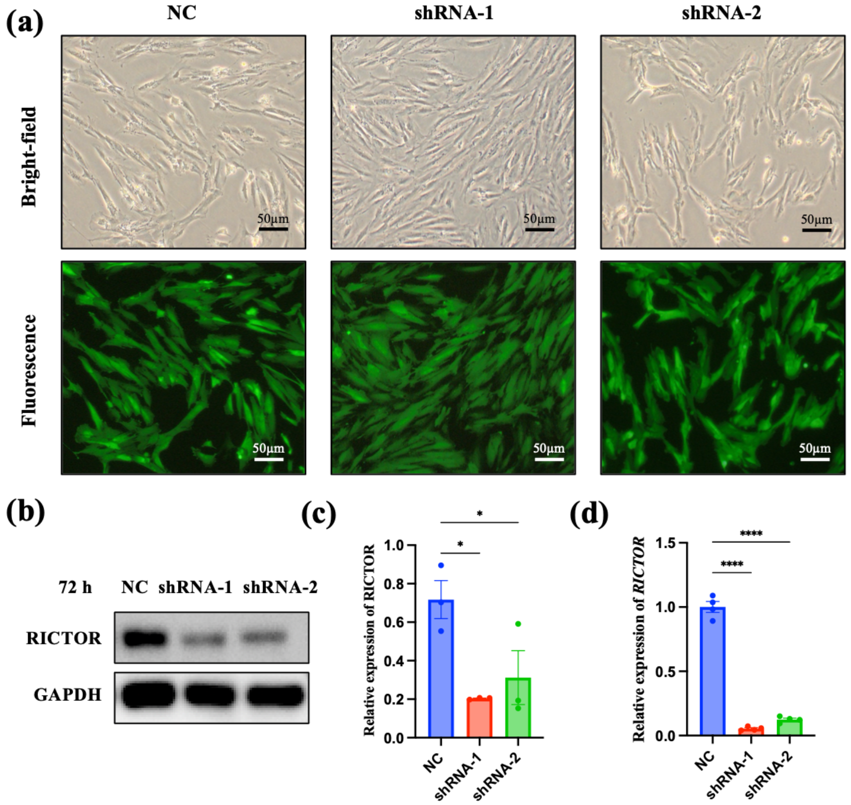

FIGURE S1 Validation of *RICTOR* knockdown efficiency via lentiviral shRNA. (a) Representative bright-field and fluorescence images of human primary endometrial stromal cells (hESCs) 72 h after transfection with NC, shRNA-1 and shRNA-2 lentiviruses. (b) Representative Western blots showing RICTOR protein expression in hESCs from young women 72h after transfection with NC, shRNA-1 and shRNA-2. (c) Quantification of RICTOR protein levels from (b), normalized to GAPDH. Data were presented as mean ± SEM, * $P < 0.05$, $n = 3$. Statistical significance was assessed using ordinary one-way ANOVA followed by Dunnett's multiple-comparisons test, with the NC group serving as the control. (d) mRNA expression levels of *RICTOR* in hESCs from young women 24h after transfection with NC, shRNA-1 and shRNA-2. Data were presented as mean ± SEM. **** $P < 0.0001$, n=4. Statistical significance was assessed using ordinary one-way ANOVA followed by Dunnett's multiple-comparisons test, with the NC group serving as the control.

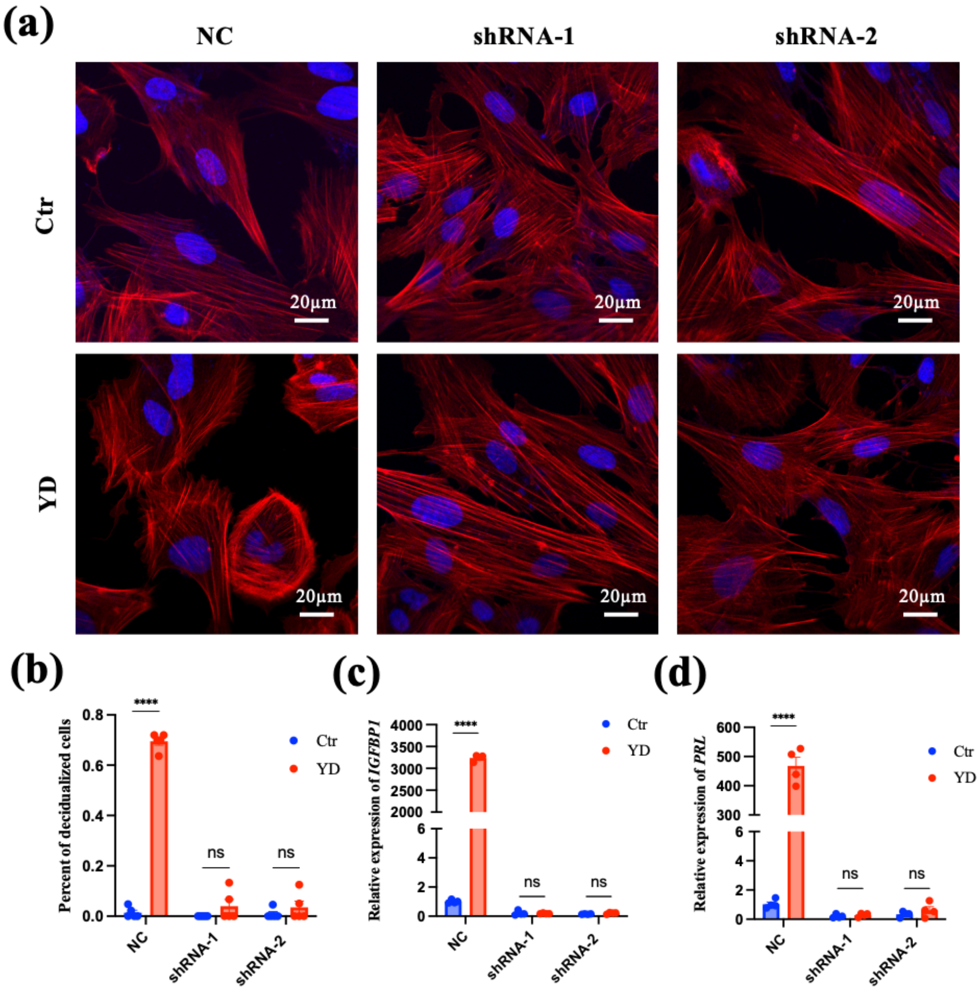

FIGURE S2 Lentiviral-mediated *RICTOR* knockdown impairs in vitro decidualization of hESCs. (a) Representative fluorescence images of hESCs following 4 days of decidualization induction. Cells were pre-transduced with NC, shRNA-1 or shRNA-2 lentiviruses. F-actin was labelled with phalloidin (red), and cell nuclei were counterstained with DAPI (blue). Ctr: untreated control; YD: decidualization induction group. (b) Quantitative analysis of the percentage of decidualized cells in (a). Data were presented as mean ± SEM. ns means P>0.05, **** P < 0.0001, n=5. Statistical significance was assessed using two-way ANOVA followed by Šídák's multiple-comparisons test. (c-d) Relative mRNA levels of decidual marker genes *IGFBP1* and *PRL* in lentivirus-treated hESCs after 4 days of decidual induction. Data were presented as mean ± SEM. ns means P>0.05, **** P < 0.0001, n=5. Statistical significance was assessed using two-way ANOVA followed by Šídák's multiple-comparisons test.

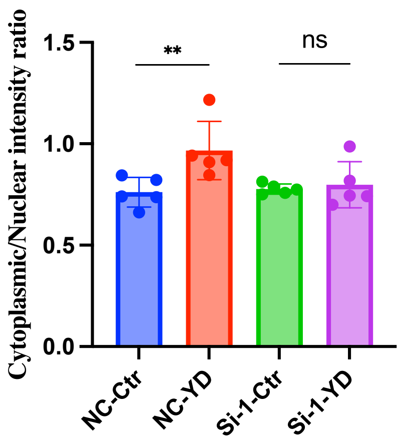

FIGURE S3 Quantification of cytoplasmic/nuclear fluorescence intensity ratio of FoxO1. Data are presented as mean ± SEM, ns means P> 0.05, * P < 0.05, n = 4. Statistical significance was assessed using two-way ANOVA followed by Šídák's multiple-comparisons test.
